# An Elementary Hand-Book on Potable Water

**Published:** 1891-11

**Authors:** 


An Elementary Hand-Book on Potable Water. By Floyd Davis, M. Sc., Ph. D.; Professor of General and Applied Chemistry in Drake University; Chemist of Iowa State Board of Health; Member of American Association for Advancement of Science; Member of American Public Health Association; Member of American Institute of Mining Engineers; Member of Wisconsin Academy of Science, Arts, and Letters; and Fellow of Iowa Academy of Sciences. Pp. 118. Silver, Burdett & Co., publishers, Boston. 1891.

This book, intended for the use of physicians, sanitarians, and chemists, differs from the majority of works on potable water, in that all analytical detail is omitted. The author gives, in a clear and concise, yet vivid and satisfactory manner, a careful discussion of drinking water, its impurities, and methods of purification.

This work shows that it comes from the pen of a chemist and sanitarian perfectly familiar with his subject, not only from the standpoint of literary attainment, but from laboratory experience as well.

The book is an excellent one, and we heartily recommend it to the earnest perusal of all sanitarians, physicians, and chemists.

